# Vocal fold paralysis in children: diagnostic and management from a case report

**DOI:** 10.1016/S1808-8694(15)31341-0

**Published:** 2015-10-20

**Authors:** Romualdo Suzano Louzeiro Tiago, Sandra Jager Patrocínio, Patricya Santos Figueiredo dos Anjos, Juparethan Trento Ribeiro, Fábio Marangoni Gil, Flávia Villin Denunci

**Affiliations:** ^1^ Ph.D. program in Otorhinolaryngology and Head and Neck Surgery under course, UNIFESP-EPM. Assistant physician, Service of Otorhinolaryngology, Hospital do Servidor Público Municipal de São Paulo; ^2^ Resident physician, Service of Otorhinolaryngology, Hospital do Servidor Público Municipal de São Paulo; ^3^ Resident physician, Service of Otorhinolaryngology, Hospital do Servidor Público Municipal de São de São Paulo; ^4^ Resident physician, Service of Otorhinolaryngology, Hospital do Servidor Público Municipal de São de São Paulo; ^5^ Resident physician, Service of Otorhinolaryngology, Hospital do Servidor Público Municipal de São de São Paulo; ^6^ Master in Speech and Hearing Pathology, PUC – Sao Paulo. Speech and Hearing Therapist, Sector of Speech and Hearing Therapy, Service of Otorhinolaryngology, Hospital do Servidor Público Municipal de Sao Paulo; Service of Otorhinolaryngology, Hospital do Servidor Público Municipal de São Paulo

**Keywords:** laryngeal paralysis, laryngeal nerves, patent ductus arteriosus

## Abstract

**V**ocal fold paralysis accounts for 10% of the larynx congenital abnormality, being the second most common cause of laryngeal stridor in childhood. As to unilateral vocal fold paralysis, the main cause is left-sided iatrogenic injury to the recurrent laryngeal nerve, secondary to surgery to correct the patent ductus arteriosus. In this study we reviewed the literature, reporting a case of a child who, after having undergone surgery to close the patent ductus arteriosus, evolved with breathing difficulty and dysphonia. We suggest that flexible fiberoptic laryngoscopy is carried out pre- and post surgery in children for whom heart surgery to correct congenital abnormalities is indicated, thus allowing for early diagnosis of vocal fold paralysis and the selection of the best management approach.

## INTRODUCTION

Vocal fold paralysis corresponds to 10% of laryngeal congenital anomalies^1^ and it presents different etiology, clinical presentation and management when comparing children and adults. Some authors observe that children vocal fold paralysis shows a slight predominance of unilateral paralysis1., 2., whereas others observed higher frequency of bilateral paralysis3., 4.. However, when the lesion is unilateral, there is higher impairment of the left vocal fold1., 2., 3., 4., 5.. It may be explained by the fact that left recurrent laryngeal nerve presents a longer path^4^, and it may be related with ductus arteriosus^2^, making it susceptible to damage during the progression of many diseases and surgical procedures. Male patients are more frequently affected as reported in many studies2., 3., 5., 6..

The main signal of vocal fold paralysis in childhood, both unilateral and bilateral, is stridor, and laryngeal paralysis is the second most common cause of stridor in this age range^2^, second only to laryngomalacia. Bilateral paralysis is normally present with cyanosis and apnea3., 4., whereas unilateral cases are presented with dysphonia and crying affections1., 4., 7..

Considering bilateral paralysis, the main causes are neurological, idiopathic and birth trauma1., 2., 3., 4., 5., 6.. Among neurological causes, Arnold-Chiari malformation, associated with meningomyocele and hydrocephalus, is the most common one and it is normally manifested at about the 3rd month of life1., 2., 3., 4.. The interval until the onset of symptoms is variable, from birth to the age of 5 years^1^. Normally, the diagnosis of bilateral paralysis is earlier than of unilateral paralysis owing to the more exuberant symptomatology of these patients^4^.

As to unilateral paralysis, the main cause is iatrogenic. Heart surgery was the main etiology found in a number of studies and in most cases, there is repair surgery of arterial canal persistence (PCA)1., 2., 4.. Zbar et al.^8^ found an incidence of vocal fold paralysis after ligation of arterial canal of 8.8%, with predominance in very low weight children (below 0.9 Kg) and pre-term children (below 26 weeks). The study suggested that as a result of modernization of neonatal techniques, we have greater survival of premature babies and a consequent increase in number of cases of PCA, with surgical indication, making it looks like the most common cause of vocal fold paralysis in children2., 8.. Other causes of unilateral paralysis are idiopathic, neurological and birth trauma^1^.

Ortner, in 1897, described the association between mitral stenosis and hoarseness related to left recurrent laryngeal nerve paralysis. In fact, Ortner's syndrome may be present in other diseases, given that the pathophysiology may be related to laryngeal nerve compression between the aorta and the pulmonary artery^9^.

The diagnosis of laryngeal paralysis in children may be made with flexible fibronasopharyngolaryngoscopy (FNL)3., 4., direct laryngoscopy^4^, and also with laryngeal ultrasound^10^. FNL has the advantage of using only topical anesthesia, and it does not affect the dynamics of the vocal folds, but it may hinder its performance in neurological patients, with abundant secretion in the upper airways, or in patients with anatomical abnormalities such as size of nasal fossa, epiglottis shape, and high and anteriorized children larynx^10^. Direct laryngoscopy allows good visualization but we should be careful when positioning the laryngoscope so that it does not cause immobilization of the vocal folds^10^. The disadvantage is the need to use general anesthesia, which can modify the movement of vocal folds depending on the anesthetic plan, but this method allows us to diagnose a possible fixation of cricoarytenoid joint, posterior glottic stenosis and vocal fold fusion4., 11..

Friedman studied the use of laryngeal ultrasound comparing it to FNL and direct laryngoscopy. The author concluded that ultrasound is a relatively safe, non-invasive, reliable, accurate and reproducible exam which can be used for the diagnosis of vocal fold paralysis. However, the exam does not replace the endoscopic exams for the diagnosis of vocal fold paralysis, serving only as a method to assess vocal fold mobility^10^.

Other exams are required to define the etiology, such as imaging exams of central nervous system, skull base, neck and thorax (computed tomography scan and magnetic resonance)^4^. The determination of type (unilateral or bilateral) and cause of paralysis is extremely important in choosing the treatment. In a child with inappropriate airway opening, tracheostomy is indicated^5^, which is a more frequent procedure in bilateral paralysis4., 5.. In patients with Arnold-Chiari malformation, the treatment of choice should be CSF shunt considering that it may revert vocal fold paralysis^5^.

The patients with paralysis caused by neurological cause have higher likelihood of recovering compared to idiopathic paralysis^1^. Spontaneous recovery of mobility happens in a significant number of cases. Rosin et al. observed 16% recovery of bilateral cases and 63% of unilateral cases^4^. Gentil et al.^3^ found 62.5% and Daya et al.^1^ noticed that patients with vocal fold paralysis secondary to neurological causes had a recovery rate of 71% and in all cases they were bilateral paralysis. Owing to the possibility of spontaneous recovery, the wait and see management is preferred by many different authors1., 3., 5., 11..

The purpose of the present study was to present a case of a child with clinical diagnosis of left vocal fold paralysis resulting from surgical treatment of arterial canal persistence as well as to discuss diagnostic aspects and management in such cases.

## CASE REPORT

BSN, female 7-year-old patient, came to the Service of Otorhinolaryngology, Hospital do Servidor Público Municipal de Sao Paulo, with history of breathy and soft voice. Twin sister, pre-term baby (31 weeks, with 1.1Kg weight), she remained in the neonatal ICU for 2 months, 30 days under mechanical ventilation with manifestation of hyaline membrane pulmonary disease, bronchopneumonia and septicemia. Ten days after discharge, she presented a new episode of bronchopneumonia and was hospitalized for 28 days, when her twin brother died of the same disease. At 4 months of age, she was hospitalized again for investigation of cardiac disease, with manifestations of tiredness and paleness when crying and during breastfeeding, diagnosed with echocardiogram as arterial canal persistence. At the age of 7 months, she was submitted to mini-thoracotomy in another hospital to have it corrected. During postoperative recovery, she progressed with respiratory failure and required tracheostomy and prolonged mechanical ventilation. After discharge, the child progressed with subglottic stenosis and was then submitted to four sessions of tracheal dilation. Up to the age of 2, she could only produce weak sounds and attempts of words. ENT assessment at the time, by flexible fibronasopharyngolaryngoscopy, suspected of left vocal fold paralysis and the patient was referred to vocal therapy, but quit treatment when moved to another state.

The current exam of the patient shows left vocal fold paralysis at lateral position with mobile right vocal fold and mild anterior subglottic stenosis that reduces subglottic space at about 30%. Her phonation is characterized by short production time and marked breathiness. On the neck, we could observe a small tracheocutaneous fistula.

## DISCUSSION

The etiology of vocal fold paralysis is quite varied and when we consider unilateral paralysis in children, the main cause is iatrogenic. In vocal fold paralysis of iatrogenic cause, the most frequently affected side is the left one, and most of the time it is secondary to surgery for PCA closure^1^.

PCA corresponds to less than 1% of congenital heart anomalies^12^, and it is more frequent in premature babies^13^. Closure of arterial canal may be induced surgically or by the use of drugs. Indometacin is a potent prostaglandin synthesis inhibitor and when administered early, it may lead to 50% success rate. If this method fails, surgery is indicated and it should be performed when the child is clinically stable or when children are asymptomatic after the age of 1 year^13^. As seen in the literature, the incidence of vocal fold paralysis after PCA correction is higher in premature and low birth weight neonates^8^. The patient reported in our case study was premature and had low birth weight, which placed her in a group with higher incidence of PCA and higher surgical risk of laryngeal recurrent nerve damage, with consequent left vocal fold paralysis. She was submitted to surgery at the age of 7 months, with mini-thoracotomy and arterial canal ligation, preventing dyspnea and stridor.

In the reported case, FNL was not performed preoperatively given that there is no routine for ordering this exam for children that are submitted to surgery for repair of congenital cardiac anomalies. We believe that this exam should be part of the pre and postoperative assessment of subjects with surgical indication for the correction of congenital cardiac anomalies, especially taking into account medical-legal aspects. Given that vocal fold paralysis is a highly frequent surgical complication in these patients, family members should be aware of the risks and sign the informed consent term.

The clinical manifestation of bilateral paralysis is formed mainly by stridor (76%), cyanosis (48%), difficulty in feeding (48%) and apnea (41%). In unilateral paralysis, we can frequently find stridor (59%), difficulty in feeding (50%), cyanosis, draft and hoarseness (32% each)^4^. In the case reported here, we observed dysphonia with marked breathiness and weak cry.

FNL and direct laryngoscopy can be used in the diagnosis, and we should give preference to the former because it is less invasive. These exams help to exclude other diseases that may be present with vocal affections and stridor, such as for example, glottic stenosis, fixation of cricoarytenoid joint and laryngomalacia1., 4.. We observed in the reported case, based on FNL, the presence of left laterilized and paralyzed vocal fold associated with mild subglottic stenosis. In the literature, we observed that airway obstruction caused by vocal fold paralysis could be exacerbated by the coexistence of other upper airway restrictive diseases, such as for example laryngomalacia, tracheobronchomalacia, subglottic stenosis and post-intubation granuloma. This association may be present in up to 45% of the cases^1^. Daya et al.^1^ observed that all children with unilateral vocal fold paralysis secondary to cardiac surgery that required tracheostomy had associated upper airway disease (subglottic stenosis or tracheomalacia).

The most widely accepted management in these cases is to wait and see, owing to the possibility of having spontaneous reversion of the paralysis, reserving tracheostomy for the cases of airways with respiratory difficulties^1^. In our case, we decided to follow up the patient, associating it with vocal therapy to reach better compensation of the right vocal fold and better voice.

When indicated, surgical intervention in cases of bilateral paralysis in paramedian position may be performed using lateralization methods of the vocal folds or reinervation of the posterior cricoarytenoid muscles. The first disadvantage is that it is not reversible, and as a result of children's growth, the final caliper of the airways may no longer be appropriate. The reinervation of posterior cricoarytenoid muscles allows opening of glottic chink, without vocal breathiness, a fact that occurs in the lateralization surgery. Moreover, we may make use of another technique in case of failure. The time required for reinervation may ranged from 2 to 6 months^11^.

In unilateral paralysis, the preferred management approach is vocal therapy. Most children have compensation and do not require surgical procedures2., 5.. Medialization techniques can be used in case of lateralized vocal fold position but these surgeries may restrict the airways. They are irreversible, and similarly to lateralization surgeries, there is difficulty to define better vocal fold positioning, owing to the fact that children are submitted to general anesthesia. In these surgeries, there is no restoration of vocal fold tension, maintaining vocal pitch control^11^. Teflon or Gelfoam injection allows immediate vocal or cough effective improvement, but it is an irreversible procedure and difficult to assess concerning the material to be injected, which may impair the voice and airway caliper^11^. Daya et al.^1^ reported a successful case and another one that had formation of granuloma and required its surgical exeresis.

## CLOSING REMARKS

We suggest the use of flexible fibronasopharyngolaryngoscopy before and after the surgery of children with the indication of cardiac surgery for the correction of congenital anomalies, allowing early diagnosis of vocal fold paralysis and definition of the intervention as early as possible.
